# Subminimal Inhibitory Concentrations of the Disinfectant Benzalkonium Chloride Select for a Tolerant Subpopulation of *Escherichia coli* with Inheritable Characteristics

**DOI:** 10.3390/ijms13044101

**Published:** 2012-03-28

**Authors:** Birgitte Moen, Knut Rudi, Erlend Bore, Solveig Langsrud

**Affiliations:** 1 Nofima-Norwegian Institute of Food, Fisheries and AquacultureResearch, Osloveien 1, N-1430 Ås, Norway; E-Mails: knut.rudi@umb.no (K.R.); erlend.bore@gilde.no (E.B.); solveig.langsrud@nofima.no (S.L.); 2 Department of Chemistry, Biotechnology and Food Science, Norwegian University of Life Sciences, P.O. Box 5003, N-1432 Ås, Norway; 3 Hedmark University College, Holsetgata 22, 2306 Hamar, Norway; 4 Nortura SA, P.O.Box 40, 4064 Stavanger, Norway

**Keywords:** *Escherichia coli*, quaternary ammonium compounds, benzalkonium chloride, selection of subpopulation, efflux pump, osmotic and oxidative stress response

## Abstract

Exposure of *Escherichia coli* to a subminimal inhibitory concentration (25% below MIC) of benzalkonium chloride (BC), an antimicrobial membrane-active agent commonly used in medical and food-processing environments, resulted in cell death and changes in cell morphology (filamentation). A small subpopulation (1–5% of the initial population) survived and regained similar morphology and growth rate as non-exposed cells. This subpopulation maintained tolerance to BC after serial transfers in medium without BC. To withstand BC during regrowth the cells up regulated a drug efflux associated gene (the *acrB* gene, member of the AcrAB-TolC efflux system) and changed expression of outer membrane porin genes (*ompFW*) and several genes involved in protecting the cell from the osmotic- and oxidative stress. Cells pre-exposed to osmotic- and oxidative stress (sodium chloride, salicylic acid and methyl viologen) showed higher tolerance to BC. A control and two selected isolates showing increased BC-tolerance after regrowth in BC was genome sequenced. No common point mutations were found in the BC- isolates but one point mutation in gene *rpsA* (Ribosomal protein S1) was observed in one of the isolates. The observed tolerance can therefore not solely be explained by the observed point mutation. The results indicate that there are several different mechanisms responsible for the regrowth of a tolerant subpopulation in BC, both BC-specific and general stress responses, and that sub-MIC of BC may select for phenotypic variants in a sensitive *E. coli* culture.

## 1. Introduction

Quaternary ammonium compounds (QACs), such as benzalkonium chloride (BC), are used in a wide range of applications such as disinfectants in food-processing and medical environments and as preservatives in pharmaceuticals and cosmetics. These antimicrobials are membrane-active agents with a target site predominantly at the cytoplasmic (inner) membrane and are also believed to damage the outer membrane of gram negative bacteria, thereby promoting their own uptake (for an overview, see [1,2]). The widespread use of these agents can lead to resistant bacteria, which may limit efficacy in pharmaceutical and cosmetic products or disinfection effects in food and medical applications [3]. There is also the concern that resistance to disinfectants contributes to antibiotic resistance [4–6], which may limit treatment options for microbial infections. There are several reports on bacteria adapted to high levels of BC and cross-resistance to other antimicrobial agents [7–10]. The involvement of efflux pumps [8,11,12] and reduction in the outer membrane porin OmpF [11,13] have been suggested as possible resistance mechanisms. In addition, changes in the LPS layer [13,14], changes in fatty acid profiles [15,16] and changes in cell surface hydrophobicity [8,17] have been suggested to influence acquisition of resistance in gram negative bacteria. Previous research reports have mainly focused on strains adapted to high concentrations of BC after serial transfer in gradually higher concentrations of BC.

In a previous study [9], we showed that after one transfer of *E. coli* in a BC-concentration 75% below MIC, the cells survived in higher concentrations in a bactericidal test. In the first transfer in the adaptation study, the MIC of BC doubled. Also, exposure of *E. coli* to salicylate, chenodeoycholate and methyl viologen increased the MIC of BC, indicating a role of general stress response systems. The mechanisms behind these responses were not determined, but in a later study, we found that *E. coli* cells exposed to BC-concentrations allowing growth had a remarkably different response from that of a range of other stress conditions [18]. While other stress factors (heat, cold, acid, alkali, salt, glycerol, ethanol and ethidium bromide) resulted in increased lag time and/or reduced growth rate, exposure to BC resulted in an initial killing of cells followed by growth at similar rates as non-stressed cells. Also, despite the major impact of BC on survival, the number of genes involved during regrowth was remarkably low compared to other stress factors. Only three genes (*b1171*, *osmB* and *ykfE* (*ivy*)) were identified as showing significantly different levels of expression (up regulated) for cells growing in the presence of BC. Gene *b1171* has no known function, *osmB* encodes a lipoprotein and *ykfE* (*ivy*) encodes an inhibitor of C-lycozyme [19,20]. As far as we know, there are no publications on the mechanisms involved in the survival strategy of *E coli* exposed to subminimal inhibitory concentrations (sub-MICs) of BC and the following growth in the presence of BC. There are two recent studies of the effect of sub inhibitory concentrations of BC in *Pseudomonas* [21,22], one was over time in biofilm and the other was a long-term continuous culture subjected to increasing levels of BC, suggesting that sub-inhibitory concentrations of BC are sufficient to select for adapted variants.

The aim of the current work was to investigate the survival mechanisms of *E. coli* growing in BC. This was done by microarray analysis (two new replicate microarray experiments performed and combined with previously published data for analysis), live-dead fluorescence microscopy, quantitative real-time PCR, knock-out strain analyses, as well as traditional growth studies. In addition, a control and two isolates selected after growth in BC were genome sequenced using the 454 (Roche) sequencing platform.

We present results showing that exposure to BC (25% below MIC) selects for a tolerant subpopulation of *E. coli* employing both BC specific and general stress responses, and that this subpopulation appears to have inheritable characteristics.

## 2. Results and Discussion

### 2.1. Morphological Changes and Cultivability as Analyzed by CFU after BC Exposure

**Figure 1:**
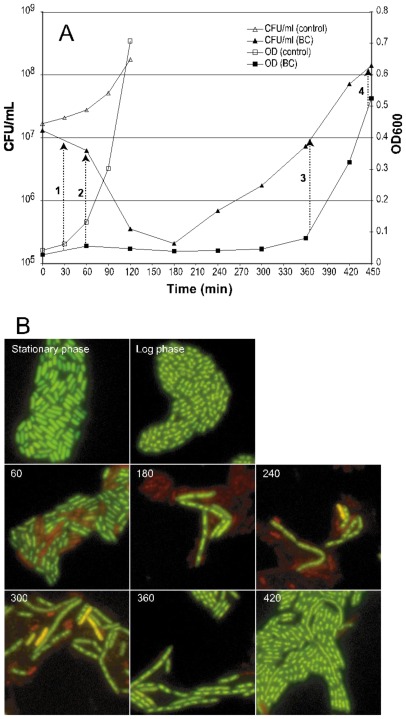
(**A**) Growth curves (CFU/mL and OD) and (**B**) *Bac*Light pictures of *E. coli* cells exposed to BC (9 μg/mL) and control at 0 (stationary phase), 60, 120, 180, 240, 300, 360 and 420 min after inoculation. Open symbols represent the control, closed symbols represent cultures with BC. The arrows and numbers 1, 2, 3 and 4 refer to the sampling points used for real-time PCR experiments.

The original MIC of BC in TSB is about 12 μg/mL [18]. Growth of *E. coli* in the presence of a BC-concentration below MIC (9 μg/mL) was analyzed by plate counts and optical density (Figure 1A), and morphological changes visualized by *Bac*Light live/dead fluorescence microscopy (Figure 1B). The addition of BC resulted in ~50% reduction of CFU/mL in the first 60 min and a total of ~98% reduction after 180 min. Exposure to BC also affected the morphology of the cells (filamentation). As also found in a previous study [18], the cells multiplied at a rate similar to the control after ~300 min and the cells regained their original morphology after 360–420 min.

To exclude the possibility that regrowth after the initial kill was due to neutralization of BC, bacteria grown in BC to mid-exponential growth phase were re-inoculated in fresh medium with BC. The regrowth times of these cells were reduced by approx. 220 min compared to cells pre-grown in medium without BC (and similar to what was observed for stationary phase control cells inoculated in TSB without BC). This indicated that the observed growth was not a result of neutralization of antibacterial action, but that the population of cells surviving exposure to BC differed from the original culture and showed higher tolerance. Regrowth of cells after the initial killing phase was delayed (compared to direct re-inoculation from BC) if the cells were grown for 1–4 transfers in medium without BC, but still shorter (approx. 150 min) than for control cells (approx. 280 min) inoculated in BC.

### 2.2. Microarray Analyses

Samples for microarray analyses were collected when the cells had regained exponential growth at a cell density of approx. 1 × 10^8^ CFU/mL (time point 4 in Figure 1). The data presented represents data from two new microarray hybridizations, BC 9 μg/mL *versus* control (no BC) (see experimental Section 3.8) in addition to a previously published experiment where only three genes (*b1171*, *osmB* and *ykfE* (*ivy*)) were identified as significantly different (up regulated) in cells grown in the presence of BC (7 μg/mL and BC 9 μg/mL) [18]. These combined data were used to identify 41 genes showing significantly different levels of expression (FDR < 0.05) in BC (9 μg/mL) compared to the control (Table 1). Among these genes were the *acrB* efflux gene, the *ompF* and *ompW* outer membrane protein genes and several genes involved in osmotic- and/or oxidative stress, in addition to several genes with unknown function. Some of these genes were studied further by knock-out mutations and real-time PCR (see Section 2.4). Among those with unknown function was gene *ykfE.* This gene has previously been shown to be a strong inhibitor of C-type lysozyme and was correspondingly renamed *ivy* [19,20]. In addition to its enzymatic activity on the bacterial cell, lysozyme also possesses a non-enzymatic inactivation mechanism. The most likely mechanism for microbial activity of lysozyme is membrane disruption as observed for several other antimicrobial peptides, rather than enzymatic degradation of the bacterial murein layer [23]. It is therefore possible that gene *ykfE* may have a function in protecting the cell against cell damaging agents.

### 2.3. Susceptibility of Knock-out Mutants

Based on difference in expression during regrowth in BC (Table 1) and availability, the role of selected genes was further investigated using single gene knock-out mutants and an expression strain. These were tested by MIC tests and regrowth tests. Regrowth time reflects the initial kill by BC and is defined as the time from inoculation to the time where the cells had reached their initial start concentration, as measured by increasing CFU and/or OD (although it cannot be excluded that a subpopulation is growing at an earlier stage). For calculation see Section 3.1. Only two (*ompC* and *ybjX*) of the 12 knock-out strains and one expression strain investigated (Table 2) showed altered regrowth time compared to the wild type when exposed to BC. Knock-out strain *ybjX* had a slight increase in regrowth time compared to the control (+ 120–150 min) and was therefore studied further in a growth competition study. The *ompC* strain had a faster regrowth (176.0 ± 22.2 min) compared to the wild type strain (254.7 ± 2.4 min) when log phase cells were used as inoculum. A delayed regrowth was also observed for knock-out strain *b1171* but this was not reproducible. These observations are discussed further under Section 2.4. The MIC of BC was similar for the knock-out strains and the wild type strain. Knock-out strains *b1171* and *ybjX* were studied further by real-time PCR and in a direct growth competition with the wild type by use of real-time PCR analysis (see Section 2.4).

### 2.4. Real-Time PCR Analyses

**Figure 2:**
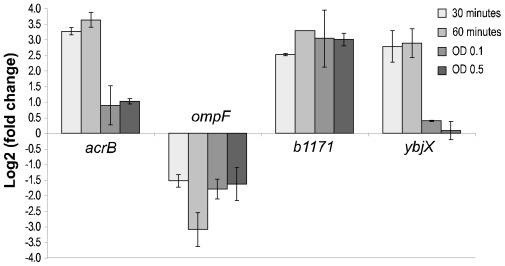
Real-time PCR results of *Escherichia coli* cells exposed to BC at 30 and 60 min, and at OD 0.1 and 0.5 after inoculation (equivalent to sampling points 1, 2, 3 and 4, respectively, shown in Figure 1). The expression is presented as log 2 of fold change compared to the reference gene, *accD*, and the control. The expression was based on 3 biological replicates.

Based on microarray- and knock-out strain results genes *acrB*, *b1171*, *ompF* and *ybjX* were studied further by real-time PCR experiments. Time points equivalent to sampling points 1–4 in Figure 1 were investigated. The early time points were chosen to investigate if the genes with altered expression during mid-logarithmic growth also were involved in the early response to BC. However, it cannot be excluded that the expression measured during these early time points could be a mixture of initial BC response and the transition from stationary growth phase to exponential growth phase. The cells grown in the presence of BC (9 μg/mL) were compared to non-stressed cells (see Section 3.5). The analyses showed that the investigated genes were also involved in the early response to BC. The expression of *acrB* in BC exposed cells was higher during the early time points than during the regrowth phase, with a maximum expression (3.6 ± 0.2) (log2 of fold change) 60 min after exposure. The expression stabilized to approx. 1.0 during the growth phase (Figure 2). A similar expression profile was observed for gene *ybjX* with maximum expression at 60 min (2.9 ± 0.5) but this gene did not seem to be up regulated during the growth phase. Gene *b1171* was up regulated at all time points during BC exposure with the highest expression at 60 min (3.3). The expression of *ompF* declined after BC exposure and was lowest at 60 min (−3.1 ± 0.5) and appeared to stabilize at approx. −1.6 during the growth phase.

The results from the real-time PCR analyses from mid-exponential growth phase (time point 4 (Figure 1), OD~0.5) were also compared to previous microarray results (OD~0.5) [18]. The results showed that the real-time PCR results coincided well with the previous microarray results for genes *acrB* and *ompF*. Gene *b1171* had a higher expression in the real-time PCR analyses, while gene *ybjX* had a lower expression. Both reference genes *accD* and *gapA* were used and gave similar results.

**Figure 3:**
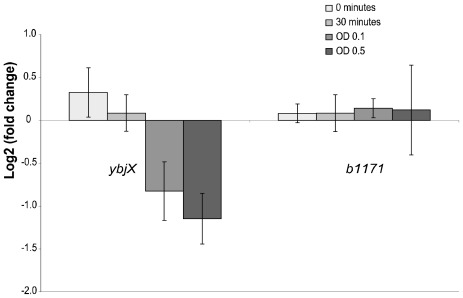
Real-time PCR results from a competitive growth experiment of mixed cultures of knock-out strain *b1171* and the wild type, and knock-out strain *ybjX* and the wild type. The time points used were equivalent to sampling points 1, 2, 3 and 4 shown in Figure 1. The log 2 of fold change was calculated using the ΔΔCT calculation based on gene *accD* and the kanamycin resistance gene related to the growth in the presence and absence of BC. The expression was based on 3 biological replicates.

Based on the results from the knock-out mutant regrowth tests in BC and the above real-time PCR results, direct growth competition tests with the wild type were performed for knock-out strains *b1171* and *ybjX*. The direct growth competition (log 2 of expression between the reference gene (*accD*) and the kanamycin resistance gene (related to growth in the presence and absence of BC), showed that the *ybjX* knock-out strain had a slower growth compared to the wild type when exposed to BC (Figure 3). The growth of the *b1171* knock-out strain in BC was not significantly different from the wild type.

The gene expression analysis showed that during regrowth in BC, the cells had a higher expression of the *acrB* gene involved in the AcrAB-TolC efflux system [25] than the control cells. The *acrB* gene has previously been linked to BC resistance [8,12] and encodes an RND (resistance nodulation cell division)-type transporter, AcrB, that together with a periplasmic accessory protein AcrA and the TolC outer membrane channel composes the AcrAB-TolC multidrug efflux system in *E. coli*. The AcrAB efflux system removes antibiotics, and other lipophilic and amphiphilic inhibitors, from the cytoplasm, through the periplasm, directly to the external media [24,25,49]. The *acrB* gene was not up regulated during growth in any of the other stress conditions [18] and we therefore believe that AcrAB-TolC contribute to survival in BC by removing BC from the cytoplasm.

Gene *ybjX* was highly expressed at 30 and 60 min after exposure to BC and knock-out analyses (direct competition growth) showed that the *ybjX* knock-out strain had impaired survival compared to the wild type when exposed to BC. This gene has no known function in *E. coli*, but showed protein homology to *somA* (S.t.) and *virK* (S.f.) *Salmonella entericia*. The *virK* gene has previously been shown to confer resistance to the cationic peptide polymyxin B, probably by contributing to remodeling of the bacterial outer membrane [50]. It is therefore possible that gene *ybjX* has a similar role of remodeling the bacterial cell envelope in response to BC in *E. coli*.

Several of the genes that showed changed expression (microarray results) during growth in BC (*hdeA*, *htrA* (*degP*), *ompF*, *ompW*, *osmB*, *pflB*, *rpoS*, *ybdQ* (*uspG*) and *yfiD*) (see Table 1 and Figure 2) have previously been associated to osmotic- and oxidative stress. This coincides with the results from our previous study where *E. coli* cells pre-cultivated in the presence of the oxidative agent salicylate resulted in higher MIC of BC [9]. The *ompF* gene was down regulated in response to BC and has previously been linked to BC resistance as well as osmotic- and oxidative stress [13,27,51]. The *ompW* (*yciD*) gene was up regulated and it has been hypothesized that OmpW might act as OmpC of *E. coli* in response to salinity stress [38]. OmpF and OmpC are major constituents of the outer membrane and are involved in osmoregulation of the cell. At low osmolarity, OmpF predominates, while at high osmolarity OmpC replaces the OmpF due to the larger pore and faster flow rate in OmpF compared to OmpC [52]. It is also known that the permeability to antimicrobial agents through the outer membrane in *E. coli* greatly depends on the contents of OmpF and OmpC porin proteins, and that OmpF deficiency makes cells resistant to some antibiotics [53–55]. Low concentrations of QACs have previously been shown to damage the outer membrane and result in the loss of many of its osmoregulatory functions [2,56]. The fact that the *ompF* knock-out strain did not show an increase in BC tolerance indicates that the observed down regulation of *ompF* was a response to increased osmolarity and not a result of preventing BC entering the cytoplasm through OmpF (as is the case for ampicillin [47,57]). This hypothesis was strengthened by the fact that the *ompC* knock-out strain (log phase) had an increased tolerance to BC, theoretically resulting in decreased expression of *ompF* as a response to increased osmolarity in addition to absence of OmpC [52].

### 2.5. Osmotic- and Oxidative Stress

**Figure 4:**
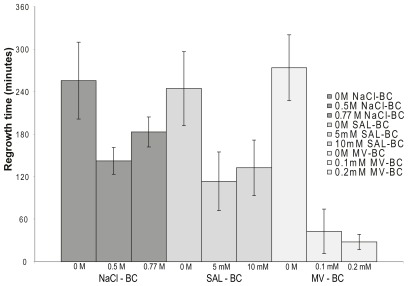
Regrowth time in BC (min) for cells pre-grown in osmotic and oxidative stress; NaCl (sodium chloride), SAL (salicylic acid), MV (methyl viologen). 0 M is the non pre-exposed control.

In a previous study [9], we showed that exposure to compounds such as salicylate and methyl viologen resulted in increased MIC of BC but no molecular studies were performed. The genetic response obtained in the present study support that common mechanisms are involved in protection against BC and osmotic or oxidative stress. To confirm previous findings and test the association with osmotic stress, growth in BC for cells pre-grown in the presence of 0.5 M and 0.77 M (4.5%) sodium chloride (NaCl): 5 mM and 10 mM salicylic acid (SAL) and 0.1 mM and 0.2 mM methyl viologen (MV) was tested. For all three compounds, the regrowth time in BC was reduced compared to cells pre-grown in pure TSB (Figure 4).

### 2.6. Characteristics of BC Isolates

**Figure 5:**
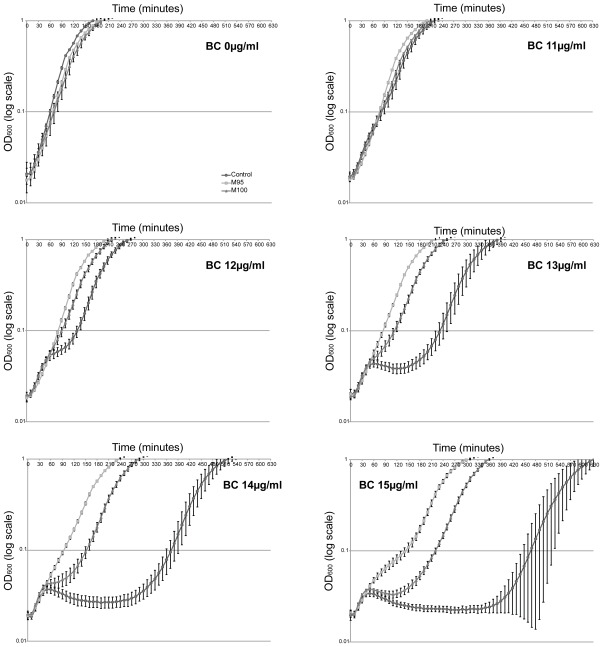
Growth curves (OD—log scale) of the Control (circle symbols), M95 (square symbols) and M100 (triangle symbols) in Bioscreen exposed to BC (0, 11, 12, 13, 14 and 15 μg/mL). The vertical bars represent the standard deviation of the ten replicates (10 wells per sample and concentration).

To investigate if the surviving subpopulation after BC exposure was a homogeneous population, a screening of 94 colonies selected after regrowth in BC were screened in Bioscreen for BC tolerance (see Section 3.11). The results showed that approx. 10% of the isolates grew better in BC than the non pre-exposed control while the remaining colonies has similar growth as the control. This indicated that the subpopulation consisted of a heterogeneous group. The two isolates with the fastest regrowth in BC compared to the control (M95 and M100) were studied further for growth in BC (see Figure 5) and selected for genome sequencing to look for common mutations. The two isolates had smaller colony morphology on TSA but had similar growth in TSB as the control. At 15 μg/mL BC the reduction in time of regrowth compared to the control was −306 and −236.7 min for M95 and M100, respectively. The isolates were also tested for maintenance of tolerance after ten repetitive overnight inoculations in medium without BC (more than 100 generations). The isolates maintained their tolerance to BC. Two random small colony variants from the control were also tested. These did not show any increased tolerance to BC, indicating that the small colony morphology itself was not responsible for increased BC tolerance.

Genome sequencing: Genome sequencing of the control and the two BC selected isolates (M95 and M100) revealed three single point mutations (in the control, M95 and M100), compared to the reference genome (*E. coli U00096*), leading to an amino acid substitution, in genes *kdpD*, *hcp* and one single point mutation in a non-coding region. A single point mutation in gene *rpsA* (nucleotide position 795 in the *rpsA* gene; T→G) leading to an amino acid substitution (Asp-265→Glu) was found exclusively in one of the BC exposed isolates (M95) (15× coverage) (see Table 3).

Ribosomal protein S1, the product of the essential *rpsA* gene, consists of six imperfect repeats of the same motif. The mutation was not in the S1 RNA binding domain. The total coverage was 13.5 for the Control, 11.6 for M95 and 12.3 for M100 given an average read length of 374.4 bp. The mutation was verified by PCR and sequencing part of the *rpsA* gene (position 683–988) in the control, M95 and M100. In addition, part of the *rpsA* gene in ten other isolates (seven that grew faster than the control and three that grew slower) selected after growth in BC was sequenced. None of these had any mutation. The RpsA protein is a target site for antimicrobial agents in the tetracycline class [58] as well as other antibacterial agents that inhibit the elongation process during bacterial protein synthesis [59,60]. The fact that the point mutation was observed in only one of the BC selected isolates, several isolates still had a higher tolerance to BC and showed altered phenotypic characteristics compared to the control, indicates that there are other additional mechanisms responsible for the observed tolerance to BC.

Based on the survival rate, the characteristics of isolates selected after BC exposure and the genome sequencing of a control and two BC exposed isolates, it is likely that BC concentrations below MIC (25% below MIC) select for phenotypic variants in a sensitive culture with the ability to survive and grow in BC. Comparable studies in *Pseudomonas*, although using a longer exposure period, have suggested that concentrations of BC below MIC are sufficient to select for adapted variants in sensitive cultures. Sub-inhibitory concentrations of BC (increasing at two-generation intervals) were shown to select for spontaneous variants of *Pseudomonas aeruginosa* grown in long-term (792 h) continuous culture [22] and adaptation over time (24 h and 96 h biofilm) of *Pseudomonas fluorescens* in biofilms to low levels of BC (10 μg/mL–50% below MIC) has been suggested by Dynes *et al.* (2009) [21]. This study shows that even short exposure to concentrations of BC allowing growth can select for BC tolerant variants of *E. coli*. The genome sequenced isolates maintained their tolerance to BC for more than 100 generations and re-inoculation study of the whole sub-population showed that the cells maintained some level of tolerance even after repetitive passages in medium without BC (approx. 13 generations). This maintenance of tolerance cannot solely be explained by the initial biomolecular- and/or gene expression stress response and appeared to be dependent on the initial selection of a subpopulation. Nor did the genome sequence of the BC isolates reveal any common mutation explaining the inheritable BC tolerance.

## 3. Experimental Section

### 3.1. Strains and Growth Conditions

The *Escherichia coli* strain used in this study was the genome-sequenced strain K12 MG1655 [61]. Bacteria were grown on tryptone soya agar (TSA, Oxoid) overnight at 37 °C. Cultures were prepared by inoculating one colony from TSA to 5 mL tryptone soya broth (TSB, Oxoid), incubation overnight at 37 °C, shaking at 200 rpm. This culture was initially diluted 1:10 and used to inoculate TSB with or without BC (dilutions from 50% BC stock solution, Norwegian pharmaceutical depot) (40–50 mL total volume) to a final concentration of approx. 1 × 10^7^ CFU/mL (1:100 dilution of overnight culture). The BC concentrations used in all experiments was 9 μg/mL (25% below MIC), except in the selection of isolates for genome sequencing, where the concentration was higher, 11.5 μg/mL, due to a new batch of BC (the stock solution used has a concentration of approximately 50% (that is 5 × 10^5^ μg/mL) according to the manufacturer). The regrowth time was nevertheless the same as for previous experiments using 9 μg/mL BC. In the Bioscreen experiments testing the two isolates chosen for genome sequencing, the BC concentrations were 11–15 μg/mL due to the new BC batch and the fact that the cells were less susceptible to BC during growth in Bioscreen plates (probably due to the smaller volume and difference in shaking regime). The lowest concentration of BC totally preventing growth after 24 h (the minimal inhibitory concentration) in TSB was 12 μg/mL, as also shown previously [18], using approx. 1 × 10^7^ CFU/mL (1:100 dilution of overnight culture) as inoculum. The knock-out strains (containing a kanamycin resistance selection gene) (Table 2), were grown overnight at 37 °C on TSA containing 50 μg/mL kanamycin (Sigma). The arabinose-inducible expression strain (*ivy*) (Table 2) was grown overnight at 37 °C on TSA containing 100 μg/mL ampicillin (Sigma) and 50 μg/mL kanamycin. Induction of *ivy* was performed by adding 0.2% (w/v) l-(+)-arabinose (Sigma) at an optic density (OD_600_) of approx. 0.2. The cells were then grown to an OD of approx. 0.5 before 5 mL of this culture was used to inoculate 45 mL TSB containing BC and 0.2% (w/v) arabinose. Growth experiments were performed with and without arabinose induction. The wild type was used as the control and grown under similar conditions as the expression strain.

The regrowth time reflects the initial killing and was defined as the time from inoculation to the time where the cells had reached their initial start concentration as measured by CFU and/or OD and estimated to be the time point where the linear regression line (Y = aX + b) equaled the optical density (OD_600_) at time zero (OD at time zero = aX + b). The linear regression line was based on three points in the linear area of the OD curve.

### 3.2. BacLight Live-Dead Fluorescence Microscopy

A 1 mL bacterial culture was centrifuged at 10,000g at 4 °C in a micro centrifuge for seven min. The supernatant was removed and the cells resuspended in 1 mL of filter sterilized peptone water. This suspension was diluted to give approximately 1 × 10^7^ CFU/mL, and 0.5 mL of this dilution mixed with 0.5 mL of the *Bac*Light (Molecular Probes) mixture (*Bac*Light ampoules mixed in 5 mL of filter sterilized water). The *Bac*Light/cell mixture was incubated for 15 min in the dark at 4 °C before being filtered down on a 0.22 micron, black polycarbonate filter (Osmonics Inc.). The filter was washed twice with 1 mL filter sterilized peptone water and then viewed by fluorescence microscopy (Leica DMLB microscope) using a RT color spot camera (Mode 2.2.0; Diagnostic Instruments, Inc.: Burroughs, Mich., USA) [62] and Spot Advanced software (version 3.0; Meyer Instruments: Houston, Tex., USA) [63].

### 3.3. RNA Extraction

Total RNA was extracted using the RNeasy Protect Bacteria Mini Prep kit (Qiagen) as recommended by the manufacturer including the “on-column” DNase treatment. The concentration and purity of the total RNA was analyzed using NanoDrop ND-1000 spectrophotometer (NanoDrop Technologies, Inc.).

### 3.4. Reverse Transcription

Total RNA was reverse transcribed by using random primers. Reaction mixture (20 μL) contained 300 ng of total RNA, 100 ng random primers (Invitrogen) and 1 μL 10 mM dNTP mix in a reaction volume of 13 μL was denatured at 65 °C for 5 min, snap cooled on ice and centrifuged briefly. The reaction mix was left at room temperature for 10 min to allow the random primers to anneal to the template. Then 4 μL 5× First Strand buffer (Invitrogen), 2 μL 0.1 M DTT was added and the reaction mix was incubated at 42°C for two min before 1 μL (200 U/μL) SuperScript II reverse transcriptase (Invitrogen) was added. The labeling reaction was incubated at 42 °C for 50 min. The reaction mix was centrifuged briefly and incubated at 70 °C for 15 min before centrifuged briefly again. Two reverse transcriptase reactions were made for each biological replicate of RNA in addition to one negative control (without enzyme). After the reverse transcriptase reaction, the cDNA was added, 20 μL water, and 2 μL of this was used as template in the quantitative real-time PCR analyses.

### 3.5. Quantitative Real-time PCR

The expression of genes *acrB*, *b1171*, *ompF* and *ybjX* were analyzed at 4 different time points (Figure 1) after inoculation in medium with and without BC (9 μg/mL); 30 and 60 min and at an OD_600_ of approximately 0.1 and 0.5. Different time points for the ΔC_T(control)_ (see data analysis) were used to calculate the ΔC_T(BC)_ at the 4 different time points. For time points 30 and 60 min, the values from the control sample at time 0 was used while the values from the control sample at OD~0.1 and OD~0.5 were used for the corresponding optical densities for cells grown in the presence of BC. The selection of the reference genes (*accD* and *gapA*) was based on the previous microarray experiment on various stress conditions [18] with the criteria that these genes did not change significantly compared to the control during the 11 stress conditions tested. In the time course experiments only gene *accD* was used as a reference gene, since the *gapA* reference gene was not an optimal choice when measuring expression during both the non-growth and growth phases.

Primers and TaqMan^®^ probes (MGB) (Table 4) were designed using Primer Express software ABI PRISM^®^ software (version 1.0; Applied Biosystems: Foster city, CA, USA) [64]. The probe contained FAM (6-carboxyfluorescein) as fluorescent reporter dye covalently linked to the 5′end, and a non-fluorescent quencher (NFQ) was covalently linked close to the 3′ end. The reporter signal was normalized to the emission of an internal reference dye (ROX-6-carboxy-X-rhodamine).

Amplification reaction mixture (25 μL) contained 2 μL template (cDNA) or 20 ng DNA; 1× TaqMan buffer A; 5 mM MgCl_2_, 200 μM each of dATP, dCTP, dGTP and dUTP; 80 μM AmpErase uracil *N*-glycosylase; 0.2 μM forward/reverse primer; 0.1 μM MGB probe; 1.25 U AmpliTaq Gold DNA polymerase (Applied Biosystems). Before amplification, the reaction mixture was heated to 50 °C for two min and then denatured at 95 °C for 10 min. The amplification profile was as follows: 40 cycles of 95 °C for 15 s and then 60 °C for one min. Reactions were performed in the Abi Prism 7900HT Sequence Detection System (Applied Biosystems). Reaction conditions were programmed and data were analyzed using the SDS software (version 2.2; Applied Biosystems: Foster City, CA, USA, 2003).

### 3.6. DNA Extraction

DNA was extracted using the DNeasy Tissue Kit (Qiagen). Briefly, 100–1000 μL bacteria was centrifuged at 5000 g for 10 min at 4 °C. The supernatant was discarded and the pellet was frozen at −20 °C for later DNA purification. The manufacture protocol was followed from this point. The concentration and purity of the DNA was analyzed using NanoDrop ND-1000 spectrophotometer (NanoDrop Technologies, Inc.) and 20 ng of DNA was used as template in the quantitative real-time PCR analyses.

### 3.7. Knock-out Strain Analyses

Knock-out strains and the expression strain were tested for altered regrowth time compared to the wild type after exposure to BC (and absence of BC). A diluted overnight culture (as described in strains and conditions) was used as inoculum in all experiments. In addition, the *ompC* and *ompF* knock-out strains were inoculated with log phase cells (OD_600_ approx. 0.5 (1 × 10^8^ CFU/mL)). Two of the knock-out strains (*b1171* and *ybjX*) were also selected for competitive growth with the wild type strain in the presence and absence of BC using quantitative real-time PCR (see Section 3.5). The wild type and the selected knock-out strains were combined in equal amounts (mixed culture) and samples were collected for purification of DNA at time zero (before addition of BC), at 30 min after exposure and at the optical density (OD_600_) of approx. 0.1 and 0.5. The two first time points were selected to capture the initial stress response to BC while the next two time points were selected to analyze initial growth (approx. 1 × 10^7^ CFU/mL) and mid-exponential growth (approx. 1 × 10^8^ CFU/mL), respectively. The method was based on the change in a reference gene, *accD*, versus the kanamycin resistance gene (KAN) inserted into the knock-out strains using the ΔΔC_T_ calculation (User Bulletin #2: ABI PRISM 7700 Sequence Detection System). In this calculation ΔΔC_T_ = [ΔC_T(BC)_ − ΔC_T(Control)_], where ΔC_T_ = C_T(KAN)_ − C_T(_*_accD_*_)_ (BC = presence of BC and Control = absence of BC).

### 3.8. Data Analysis

Microarray data from cells exposed to BC (7 and 9 μg/mL) (previously published data set [18]) was used to create gene lists of the 50 genes with lowest false discovery value (FDR) from BC (7 μg/mL), BC (9 μg/mL) and BC (7 and 9 μg/mL). In addition, two new replicate microarray hybridizations were performed (direct comparison between the control and BC (9 μg/mL)) (method as previously described [18]) identified 19 genes that were either up- or down regulated compared to the control. These new 19 genes were combined with the other 50 genes to generate a new gene list of 69 genes. These genes were present in all the observations (hybridizations) from the previous microarray experiments [18]. These 69 genes were used as responses in a new 50–50 MANOVA analysis of the stress experiment previously described [18] and resulted in 41 genes, showing a statistically significant (FDR < 0.05) difference in expression level between BC (9 μg/mL) and the control (Table 1). This list of significant genes was used to select genes for knock-out analyses and real-time PCR analyses. The ΔΔC_T_ calculation (User Bulletin #2: ABI PRISM 7700 Sequence Detection System) was used to calculate the changes in gene expression compared to the reference genes (*accD* and *gapA*) and the control sample (ΔΔC_T_ = [ΔC_T(BC)_ − ΔC_T(control)_], where ΔC_T_ = C_T(_*_test gene_*_)_ − C_T(_*_reference gene_*_)_). The expression is reported as log 2 of fold change (2^−ΔΔC_T_^). For the ΔΔC_T_ calculation to be valid, the efficiency of the target amplification and the efficiency of the reference amplification must be approximately equal. This was tested and all primer/probe pairs had an amplification effect higher than 95%.

### 3.9. Pre-Exposure to Osmotic- and Oxidative Stress Experiments

Cells were exposed to 0.5 M and 0.77 M sodium chloride (NaCl): 5 mM and 10 mM salicylic acid (SAL) and 0.1 mM and 0.2 mM methyl viologen (MV) to simulate osmotic- and oxidative stress. The cells were inoculated as described in strains and growth conditions and grown to approx. 1 × 10^8^ CFU/mL before being inoculated to fresh medium (containing BC) (1:10 dilution of the inoculation culture). The results are presented as the observed regrowth time in BC.

### 3.10. Genome Sequencing (454 (Roche) Sequencing Platform)

Selection of isolates: Cells exposed to 11.5 μg/mL BC (resulting in same regrowth time as previously using 9 μg/mL) were grown to OD~0.5 and dilutions were plated on TSA and incubated overnight at 37 °C. From these plates 94 individual colonies were picked (in addition to 3 colonies from the control grown without BC and 3 negative controls (medium only)) and transferred to a 100 well-format Bioscreen C (MTX Lab Systems, Inc.) (350 μL TSB total volume) for overnight growth at 37 °C with shaking. Cultures from this plate were initially diluted 1:10 and used to inoculate TSB without or with BC (12 μg/mL) in 2 new 100 well-format plates for growth and on-line turbidity measurement (10 wells per sample and concentration). Based on the results, glycerol stocks were prepared for a selection of the 94 colonies and stored at −80 °C. One control sample (Control) and two samples from growth in BC (M95 and M100) were tested again for growth in BC before being selected for genome sequencing. A small amount from the freeze stocks were plated on TSA for overnight growth at 37 °C. One colony from each sample were transferred to 5 mL TSB and grown at 37 °C overnight and tested for growth in Bioscreen (5 different BC concentrations 11, 12, 13, 14 and 15 μg/mL (all concentrations were below MIC in Bioscreen using a new batch of BC)). There were 10 replicate wells per isolate and BC concentration. DNA was purified from the overnight growth in TSB using the Qiagen Blood and Tissue kit (Qiagen). Briefly, 2 500 μL bacteria were centrifuged at 5000 g for 10 min at 4 °C. The supernatant was discarded and the pellet was frozen at −80 °C for later DNA purification. The manufacture protocol for gram-positive bacteria was used in addition to lysozyme in the pretreatment buffer and an RNase step. The concentration and purity of the DNA was analyzed using NanoDrop ND-1000 spectrophotometer (NanoDrop Technologies, Inc.) in addition to EtBr-stained 1% agarose gel containing 500 ng of each sample. The DNA was sent to the Norwegian High-Throughput Sequencing Centre (NSC) (University of Oslo) for genome sequencing using the 454 (Roche) sequencing platform.

Data analysis: The sequence reads were assembled and analyzed using the CLC Genomic Workbench software (version 3.7.1; CLC Bio.: Aarhus, Denmark, 2010). The three isolates, Control, M95 and M100, were all assembled against the *E. coli* U00096 reference genome. The SNP Detection tool (using default parameters) was then used to search for single point mutations.

## 4. Conclusions

There are most likely several interacting mechanisms involved in the response to BC at concentrations allowing growth, both BC specific and general stress related, and the apparent inheritance of tolerance to BC in *E. coli*. In summary, we have shown that exposure to a BC concentration 25% below MIC in nutrient medium results in initial killing of 95–99% of the cells followed by regrowth of a more BC tolerant subpopulation of *E. coli* in a sensitive culture and that this tolerance is partly inheritable. To withstand BC during regrowth, genes responsible for removing BC from the cytoplasm, the AcrAB-TolC efflux system, and genes involved in maintenance of the outer membrane in response to increased osmotic- and oxidative stress are important. Genome sequencing of a control and two selected isolates showing increased BC-tolerance after growth in concentrations of BC 25% below MIC identified no common point mutations in the BC tolerant isolates. One point mutation in gene *rpsA* (Ribosomal protein S1) was observed in one of the isolates. The observed tolerance can therefore not solely be explained by the observed point mutation. Further work is needed to address the mechanisms behind the inheritance of tolerance to BC and the possible role of the observed single point mutation in gene *rpsA*.

## Figures and Tables

**Table 1:** Genes significantly different (FDR < 0.05) in BC (9 μg/mL) compared to the control (based on two new microarray experiments and data from a previous experiments [18]) and corresponding *t*-values, expression (log 2 compared to the control) and description.

Function and Gene Name	Log 2 (BC/Control)	Gene Description and References
**Efflux System**		

*acrB*	0.9	AcrAB-TolC efflux pump [24,25]. The *acrB* gene has previously been linked to BC resistance [8,12].

**Osmotic and/or Oxidative Stress**		

*hdeA*	1.6	The *hdeA* gene encodes the acid stress chaperone HdeA that enhances survival in extreme acid conditions [26]. The *hdeA* gene has also been shown to be induced by sodium salicylate [27].
*htrA* (*degP*)	0.8	Membrane-associated serine endoprotease (known heat shock protein that has also been shown to be induced by ampicillin and kanamycin in *E. coli* [28] and are hypothesized to participate in the degradation of oxidatively-damaged proteins localized in the cell envelope [29,30].
*ompF*	−1.7	The OmpC and OmpF porins account for approx. 2% of the total protein content of the cell [31] and allow for the passive diffusion of solutes across the outer membrane. *ompF* has previously been shown to be regulated by changes in osmolarity [32] and in response to oxidative stress [27] and has been linked to BC resistance [13].
*osmB*	1.6	*osmB* is a multistress-responsive gene and encodes an outer membrane lipoprotein of unknown function [33]. *osmB* is transcribed under the control of two independent promoters, one is responsible for the response to the growth phase and to osmotic shock, whereas the other is the target of response regulator RcsB [34] which is a positive regulatory gene for capsule (colanic acid) synthesis.
*pflB*	1.1	Pyruvate formate lyase I. Gene *pflB* has previously been shown to be induced by sodium salicylate [27].
*rpoS*	0.8	The *rpoS* gene (σ^S^) acts as the master regulator of stationary phase response and general stress response [35] and controls the expression of genes involved in (among others) osmotic- and oxidative stress [36].
*yciD* (*ompW*)	2.2	Gene *yciD* (or *ompW*) encodes an outer membrane protein (OMP) and mutants that lacked the OmpW protein have been shown to be resistant to colicin S4 [37]. Studies on *Vibrio alginolyticus* [38] have shown the up regulation of an OmpW homolog in response to high osmolarity (NaCl) and it was hypothesized that OmpW might act as OmpC of *E. coli* in response to salinity stress.
*ybdQ* (*uspG*)	1.1	The *ybdQ* (or *uspG*) gene share homologies with UspA and have been shown to accumulate under various growth inhibitory conditions and induced by heat shock and may function as a universal stress protein [39]. Gene *ybdQ* has previously been shown to be up regulated in response to osmotic upshift in *E. coli* [40].
*yfiD*	2.0	Glycine radical cofactor that can reactivate pyruvate formate lyase after oxidative stress. Gene *yfiD* has been shown to be up regulated during oxidative stress and was hypothesized to function in protecting bacterial cells from oxidative stress [41,42].

**Other**		

*b2097* (*fbaB*)	1.0	Fructose 1,6-bisphosphate aldolase
*edd*	−2.1	Phosphogluconate dehydratase
*eutH*	−0.9	Putative integral membrane protein
*fruB*	1.0	Fructosephosphotransferase enzyme III
*gntK*	−3.3	Genes *gntKTU* constitute the GntI system and metabolize gluconate via the Entner-Doudroff pathway [43]. *gntT* and *gntU*, encodes a high- and low- affinity gluconate transporters, respectively, and *gntK*, encode a thermo-resistant gluconokinase.
*gntT*	−3.1	
*gntU_1*	−3.1	
*hdhA*	0.6	7-alpha-hydroxysteroid dehydrogenase
*lrhA*	−1.0	Gene *lhrA* encodes a LysR-type regulator LrhA [44] a new transcriptional key regulator of flagella, motility and chemotaxis genes in *E. coli*. LrhA is also shown to function as a regulatory component in the RpoS-dependent growth phase repression of *ompF* [45].
*malZ*	0.7	Maltodextrin glucosidase
*nagE*	−0.6	*N*-acetylglucosamine-specific enzyme II of phosphotransferase system
*nirD*	3.5	Nitrite reductase [NAD(P)H] subunit
*plsB*	0.7	Glycerolphosphate acyltransferase activity
*rbsB*	1.5	d-ribose binding protein, periplasmic
*rplN*	−0.4	50S ribosomal subunit protein L14
*speB*	−0.6	Agmatinase
*tktA*	0.4	Transketolase; binds Zn(II)
*torS*	−0.7	Sensor kinase for torCAD operon
*tpiA*	0.8	Triosephosphate isomerase
*ybbU* (*allR*)	0.7	Repressor for allantoin (all) and glyoxylate (gcl) utilization operons
*yecI* (*ftnB*)	1.2	Ferritin-like protein, function unknown
*yhiW* (*gadW)*	1.7	Positive AraC-type regulator of *gadA* and *gadBC*
*yihG*	0.6	Characterized as poly(A) polymerase II, but this claim has been contradicted
*ykfE* (*ivy)*	1.4	The *ykfE* gene has previously been shown to be a strong inhibitor of C-type lysozyme and was correspondingly renamed *ivy* [19,20].

**Unknown**		

*b1171*	2.1	Gene *b1171* (or *ymgD*) has no known function and produced no hit on a Pubmed search. The protein sequence produced no close hits other than a 100% identity to a hypothetical protein in *Shigella flexneri* (BLAST).
*b2107*	1.0	Function unknown
*b2295*	0.5	Function unknown
*yaiL*	0.8	Function unknown
*ybjX*	0.6	Gene *ybjX* has no known function but has 99% protein homology to a putative enzyme in *Shigella flexneri* 2a and 55% protein homology to a putative VirK protein in *Salmonella enterica* (180 identical of 330 amino acids) (BLAST).
*yieE*	1.0	Function unknown
*yieF*	0.8	The *yieE* gene has no known function but showed 97% identity (246 identical of 253 amino acids) to a hypothetical protein from *Shigella flexneri* and 77% identity (194 identical of 253 amino acids) to a putative cytoplasmic protein from *Salmonella typhimurium* (BLAST).

Gene description is derived from the cited references and from EcoGene [46].

**Table 2:** Knock-out strains and expression strain used in this study.

Strain	Knock-Out Strain Details a	Source or Reference
MG1655	*b1171*::Tn5KAN-I-SceI	University of Wisconsin, Madison b
MG1655	*gntK*::Tn5KAN-I-SceI	University of Wisconsin, Madison b
MG1655	*gntT*::Tn5KAN-I-SceI	University of Wisconsin, Madison b
MG1655	*hdeA*::Tn5KAN-I-SceI	University of Wisconsin, Madison b
MG1655	*lrhA*::Tn5KAN-2	University of Wisconsin, Madison b
MG1655	*ompC*::Tn5KAN	[47]
MG1655	*ompF*::Tn5KAN	[47]
MG1655	*osmB*::Tn5KAN-2	University of Wisconsin, Madison b
MG1655	*ybjX*::Tn5KAN-I-SceI	University of Wisconsin, Madison b
MG1655	*yciD*::Tn5KAN-I-SceI	University of Wisconsin, Madison b
MG1655	*yieF*::Tn5KAN-I-SceI	University of Wisconsin, Madison b
MG1655	*ivy*::KAN	[19]
MG1655	pAA410-arabinose-inducible Ivy overexpression strain	[19]

a KAN, kanamycin resistance;

b *E. coli* Genome project [48]. Published by Deckers *et al*., 2004 [19].

**Table 3:** Single point mutations identified in the Control, M95 and M100 when assembled with the *E. coli U00096* reference genome.

Allele Variation	Single Point Mutations (+/−)			Reference Position	Gene Annotations	Amino Acid Substitution

	Control	M95	M100			
G→T	+	+ a	+ a	720994	Gene: *kdpD*	Gln→Lys
A→G	+ a	+	+	911614	Gene: *hcp*	Leu→Pro
C→T	+	+ a	+	3957957	No coding region	-
T→G	− b	+	− b	962012	Gene: *rpsA*	Asp→Glu

a The single point mutations were not reported when using default parameters, but were verified by visual inspection (the lowest coverage was seven);

b Visual inspection confirmed the absence of the single point mutation in the Control and M100. This was also verified by sequencing.

**Table 4:** Primers and fluorogenic probes for real-time PCR analyses.

Gene	Probe or Primers	Sequence (5′-3′)	Denaturation Temp. (°C)
*accD*	probe	CGCAGTGAATTCC	70
	forward primer	TGCCGCCTGGATTCCA	59
	reverse primer	GTCGATCGCGCCTTTCTC	58
*gapA*	probe	CGAAACTGCTCGTAAAC	69
	forward primer	AAGCAACTGGTCTGTTCCTGACT	58
	reverse primer	TTCGCACCAGCGGTGAT	58
*acrB*	probe	TGCGATGGTTTTCG	69
	forward primer	GCGCTTTCTCGCAAATCAA	59
	reverse primer	CGATTGCGGGCAGGTTAA	59
*ybjX*	probe	CATCGCCGTAGTTTT	68
	forward primer	TCGGCCGGGTAAATTCTG	58
	reverse primer	CGAGCGCAGCAAAAATTTCT	59
*b1171_ymgD*	probe	CCCGCAAATGC	68
	forward primer	ACTGAATCAGGTTTGCGCTAAAG	59
	reverse primer	TCATCAATTGCCGTGATCAAC	58
*ompF*	probe	ACCTGGGTAAAAACGA	68
	forward primer	TGGCCTGAACTTCGCTGTT	58
	reverse primer	GAACGGCGTGCAGTGTCA	59
KAN	probe	TGCGCCGGTTGC	69
	forward primer	TTGATGCGCTGGCAGTGT	58
	reverse primer	GGACAATTACAAACAGGAATCGAA	58

All probes were MGB probes (5′ label: 6FAM and 3′ label: MGBNFQ (Minor groove binder/Non-fluorescent quencher).
